# On the Seismic Performance of Autoclaved Aerated Concrete Self-Insulation Block Walls

**DOI:** 10.3390/ma13132942

**Published:** 2020-06-30

**Authors:** Yun Liu, Gonglian Chen, Zhipeng Wang, Zhen Chen, Yujia Gao, Fenglan Li

**Affiliations:** 1 School of Civil Engineering and Communication, North China University of Water Resources and Electric Power, Zhengzhou 450045, China; liuyun@ncwu.edu.cn (Y.L.); chengonglian@ncwu.edu.cn (G.C.); chenzhen@ncwu.edu.cn (Z.C.); 2 International Joint Research Lab for Eco-Building Materials and Engineering of Henan, North China University of Water Resources and Electric Power, Zhengzhou 450045, China; wh@ncwu.edu.cn; 3 The Fourth Construction Co., Ltd. of CSCEC 7th Division, Xi’an 710016, China; gaoyuj23@cscec.com

**Keywords:** autoclaved aerated concrete (AAC), self-insulation block, masonry wall, seismic behavior, shear capacity, hysteresis behavior

## Abstract

Autoclaved aerated concrete (AAC) self-insulation block masonry is often used for the infill walls in steel and concrete frame structures. To work together with the frame under earthquake action, it is essential to understand the seismic behavior of AAC self-insulation block masonry walls. In this paper, six AAC self-insulation block masonry walls were experimentally studied under the pseudo static test. The load-displacement hysteretic curves were drawn with the test data. The failure characteristics, loading capacity, stiffness degeneration, energy dissipation capacity and hysteretic behavior are analyzed. The results indicate that the blocks underwent internal failure due to the lower strength with a larger size, but the walls had good energy dissipation capacity with a rational bearing capacity. Accompanied by the influence of vertical compressive stress on the top surface of the walls, the cracking resistance, ultimate bearing capacity, deformability and energy dissipation capacity of the walls were affected by the masonry mortar joints. Comparatively, the walls with thin-layer mortar joints had better seismic performance than those with insulation mortar joints or with vertical joints filled by mineral wool plates. Finally, the shear capacity of the walls under seismic load is evaluated referring to the formulas of current design codes for masonry walls.

## 1. Introduction

Autoclaved aerated concrete (AAC) is a type of green building material to be used for the energy conservation and fire-proofing of buildings [1,2,3]. Because of its features, namely that it is self-lightweight and has lower modulus of elasticity of the AAC, the steel and reinforced concrete frame structures with infill AAC block masonry walls have good earthquake resistance to diagonal cracking, corner crushing and severe damage states [4,5,6].

The studies on seismic performance of AAC block masonry walls indicate that a limited in-plane displacement capacity of the walls was strongly dependent on the applied vertical load, and a residual vertical strength in the order of 40–50% of the initial load-bearing capacity benefited the post-earthquake safety [7,8]. The presence of the infill walls clearly produced an additional shear demand along the contact length of the reinforced concrete columns; the contact length increased from about 30% to more than 50% of the column clear height with the imposed displacement. This leads to the significant differences in the distribution of the internal forces between the bare and the infilled frames [9].

To enhance the entirety of the AAC block masonry walls, several measurements were proposed and experimentally verified. The fiber mesh plaster layer could improve the serviceability of the infill walls at low deformation demand due to the elimination of the visible cracking [10]. With the confinement of horizontal and/or vertical reinforcements, the walls exhibited a general improvement of the displacement capacity and the reduction of damage subjected to horizontal actions. The presence of flat-truss bed-joint reinforcement in horizontal joints allowed an increase in strain and dissipative capacity of the wall, and a limitation of the damage in terms of spreading and extent of cracks. This provides a substantial improvement in the overall seismic performance of the wall with increased maximum deformation capacity and shear strength [11,12]. By placing the glass-fiber mesh in the horizontal mortar joints, the walls were improved in seismic performance with better cracking resistance, higher capacity resisting horizontal action and displacement, and a damage changing from brittle to ductile [13]. Compared with the bare frame, the infilled frames increased respectively in the yield load, the maximum load and the ultimate load by 31–159%, 51–156% and 45–123%. In the condition of being disconnected or flexibly connected with columns, the infill walls slightly influenced the seismic behavior of reinforced concrete frame including the bearing capacity, the deformation, the stiffness and the energy dissipation [14].

With the development of energy-efficient buildings, an innovative series of high-efficiency thermal-insulation materials for enveloping building walls have been created. One of them is the AAC self-insulation block. Based on previous studies, the AAC self-insulation block has features of much lower thermal conductivity under the premise of ensuring rational mechanical properties [15]. Meanwhile, the thermal insulation mortar used for AAC block masonry was also developed [16,17]. This provides a favorable construction for the enveloping walls of buildings without any other heat preservation. Because they are new products for building construction, there is a lack of study on the seismic performance of AAC self-insulation block masonry used for the infill walls. To fill this gap and accumulate reliable data for design standard, this paper arranged an experimental study of six AAC self-insulation block walls under the pseudo static test. The crack developing and failure characteristics were observed, and the load-displacement hysteretic curves were measured. The test results are analyzed in detail, and the shear capacity of the walls under seismic load is evaluated referring to the formulas of current design codes for masonry walls.

## 2. Experimental

### 2.1. Properties of AAC Self-Insulation Blocks and Mortars

The self-insulation AAC blocks were made by Henan Xing’an New Building Materials CO., LTD. The pulverized coal ash slurry, plaster paste, ordinary silicate cement, aluminum paste and foam stabilizer were used as the raw materials. Specific mix proportion was designed based on the manufactured technics. The dimension of block was 600 mm long, 300 mm height and 250 mm wide. Based on the tests of the blocks [15], the dry density was 558 kg/m^3^, the compressive strength was 4.1 MPa, the water absorption was 63.5%, and the thermal conductivity was 0.11 W⁄(m·K). As per the specification of China code GB 11968 [1], the block belongs to the superior product with class of strength A5.0 and density B06.

Two kinds of masonry mortar were used for the construction of wall specimens [15]. One was the thin-layer mortar for the joints with thickness of 5 mm. It was prepared by the market supplied dry-mixed mortar to water with mass proportion of 1:0.48. Another was the insulation mortar for the joints with thickness of 10 mm. It was made of the market supplied dry-mixed mortar admixed with expanded perlite and vitrified microsphere. The mass proportion of dry-mixed mortar: expanded perlite and vitrified microsphere: water was 1:0.15:0.46. The compressive strength of the thin-layer mortar and the insulation mortar was 17.1 MPa and 8.7 MPa, respectively.

The basic mechanical performances of AAC self-insulation block masonry were measured as per China code GB/T 50129 [18]. The test methods for the compressive strength and the shear strength along joints of the AAC self-insulation block masonry are concretely presented in related researches reported previously [15]. The masonry with thin-layer mortar joints had a compressive strength of 1.99 MPa and a shear strength along joints of 0.25 MPa. The masonry with insulation mortar joints had a compressive strength of 1.84 MPa and a shear strength along joints of 0.37 MPa.

### 2.2. Preparation of Wall Specimens

**Figure 1:**
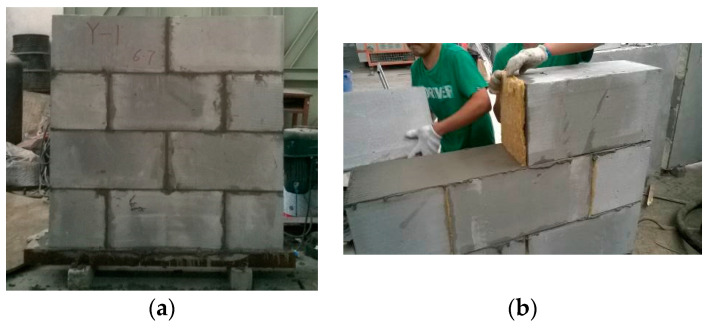
Construction of wall specimens: (**a**) overview; (**b**) vertical joints filled with mineral wool plate.

As presented in Figure 1, six wall specimens were made and tested for proving the seismic behaviors. Two of them were built respectively as a group with the self-insulation mortar joints, the thin-layer mortar joints and the horizontal thin-layer mortar joints accompanied with vertical mineral wool plate joints. The mineral wool plate was mainly produced by molten basalt material.

Before seismic testing, all specimens were tested to measure the heat transfer coefficient to gather the test data. The thermal-insulation test method was reported in previous studies [15,19]. As presented in Table 1, the heat transfer coefficient of block masonry is almost the same as previous tests. This indicates the steady production quality of the AAC blocks.

After the testing of heat transfer coefficient, the bottom surface of the wall specimen was bonded on steel basement by epoxy resin adhesive in order to carry out the seismic experiment.

Based on the engineering application of the AAC self-insulation block masonry used for the infill walls of frames, two levels of vertical compressive stress at 0.3 MPa and 0.5 MPa were applied on the top surface of the walls. This was used to examine the effect of vertical compression on the seismic performance of the walls.

### 2.3. Seismic Test Method

**Figure 2:**
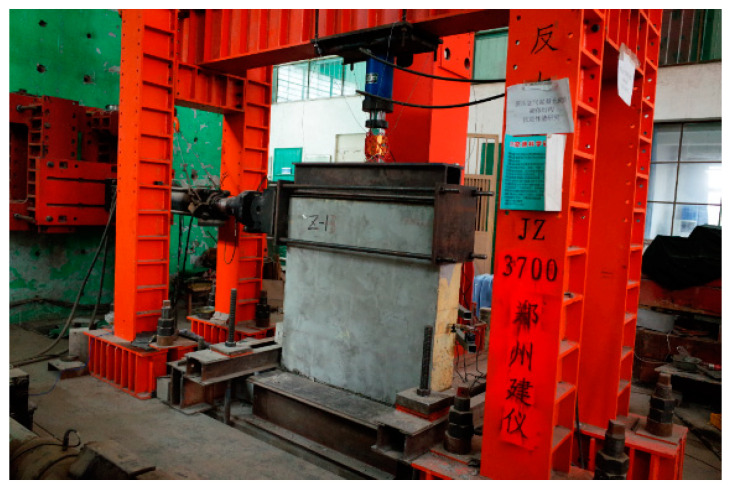
Scene photos of test devices and wall specimen.

As per China code JGJ/T101 [20], the testing apparatus is exhibited in Figure 2. The steel basement of the specimen was fixed on the foundation by ground anchorages. The horizontal cyclic load was exerted by an actuator fixed on the reaction wall, and transferred to the head of specimen by a steel hoop. The actuator was made by MTS Co. Ltd., Minneapolis, MN, USA. The vertical load was exerted by a hydraulic jack accompanied with a load sensor, and distributed uniformly with a steel beam on the top surface of specimen. Rollers were set between the steel beam and the top surface of specimen to provide freely horizontal displacement of the head of specimen. The horizontal cyclic loads and the vertical load were automatically operated by a computer.

A group of displacement sensors placed at the sides and the basement of specimen. The data were automatically collected by a data acquisition system. Based on the test data, the horizontal displacement at the head of the specimen can be computed relatively to the basement.

When ready for the formal test process, the vertical load was exerted continuously to the value controlled by the vertical compressive stress presented in Table 1. In the whole process of test, the vertical load was maintained as constant.

**Figure 3:**
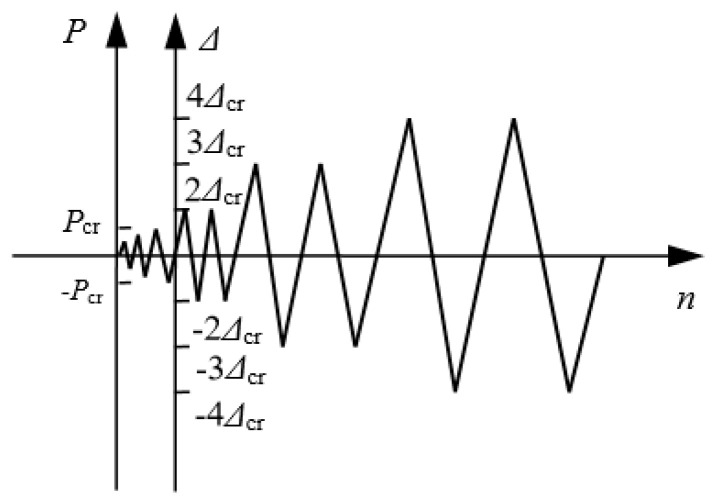
Horizontal loading program of pseudo-static test.

After that, the horizontal loading procedure was performed according to the load-displacement dual control method as presented in Figure 3. The horizontal load was graded on the wall before cracking. The grading was reduced to catch the cracking resistance when the load was close to the predictive cracking load. After cracking of the wall, the horizontal load was exerted by the displacement. At values of two and three times the cracking displacement, the load was applied for two cycles, respectively. Then, until damage was caused, the controlled displacement was four times the cracking displacement. When the bearing capacity at push/pull directions reduced to 85% of the corresponding ultimate loads, the wall was regarded to be damaged, and the test was over.

## 3. Test Results and Discussion

### 3.1. Crack Distribution and Failure Pattern

**Figure 4:**
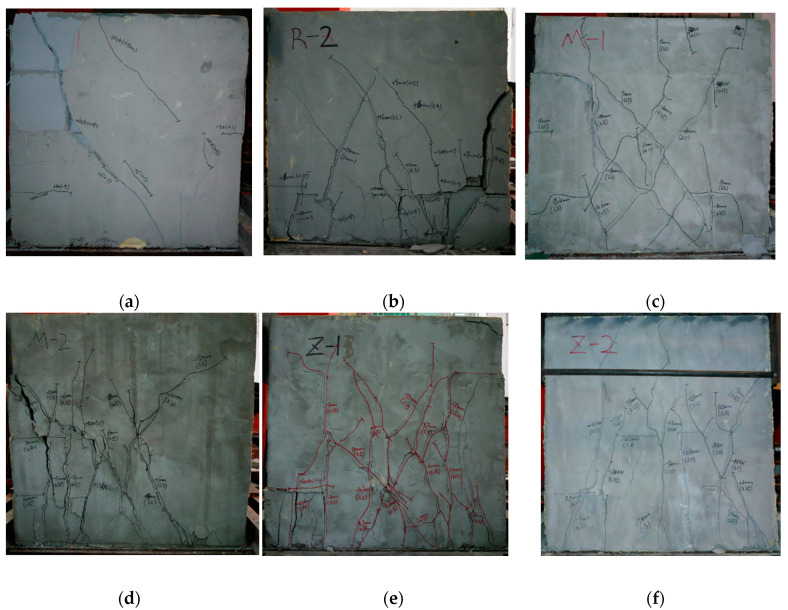
Failure modes of walls: (**a**) R-1; (**b**) R-2; (**c**) M-1; (**d**) M-2; (**e**) Z-1; (**f**) Z-2.

Figure 4 presents the crack distribution and failure patterns of the walls. Before cracking, almost no residual displacement was exerted after each cycle of the load. The slant crack appeared at about 60% ultimate load while the displacement on top of the wall was about 2 mm. With the increase of load, the residual displacement of the walls after each cycle of load accumulated, and the cracks developed to be intersected together. The damage happened with the cracks along the horizontal joints. A typical failure pattern with slant-intersected cracks appeared on the walls with thin-layer mortar joints, as presented for the walls of M-1 and M-2. This shows more internal damage of the AAC blocks. Relatively more vertical cracks appeared on the walls Z-1 and Z-2. This means that a larger horizontal tensile stress took place on the walls to break the plastering surface of AAC blocks due to the non-bonded vertical joints filled with mineral wool plates. Under higher vertical compressive stress of 0.5 MPa, for the instance of R-2 and M-2, the blocks on the push/pull sides of the walls were easily broken without confinement.

### 3.2. Load-Displacement Hysteretic Curves and Featured Values

**Figure 5:**
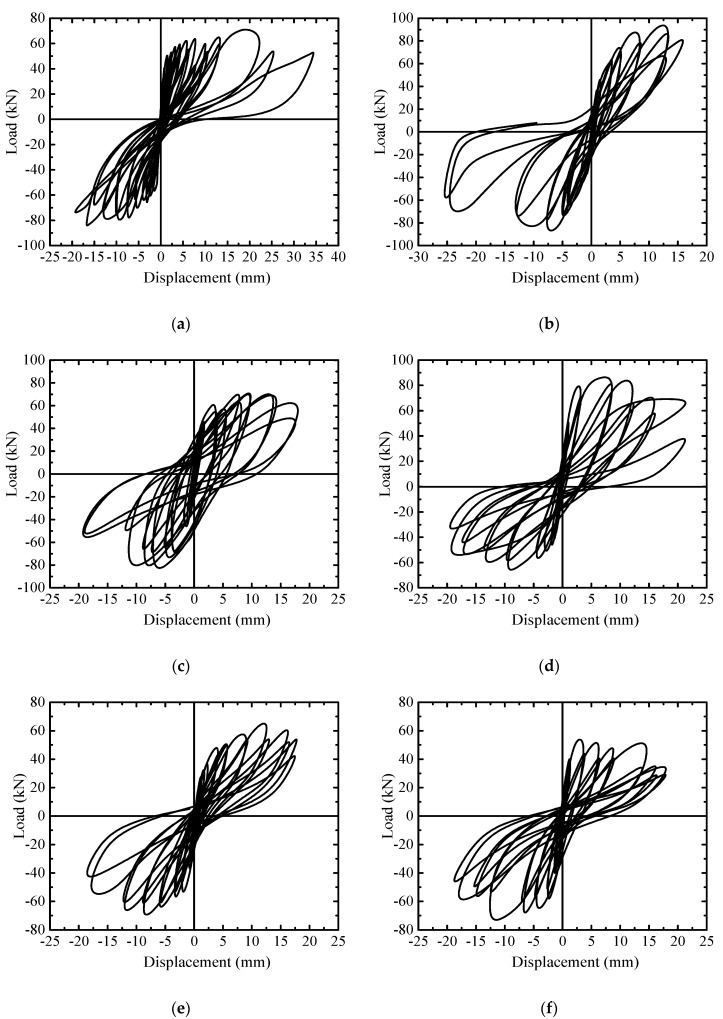
Hysteretic curves of wall specimens: (**a**) R-1; (**b**) R-2; (**c**) M-1; (**d**) M-2; (**e**) Z-1; (**f**) Z-2.

The load-displacement hysteretic curves of specimens are presented in Figure 5. Combined with the crack distribution and failure pattern of the walls, the curves exhibit the following features. Before cracking, the curves are nearly linear without residual displacement at the end of each cycle, the walls worked in an elastic manner with similar stiffness. After cracking, the curves went outward with increased hysteresis area, and appeared to be fusiform. With increased horizontal load, the walls worked into an elasto-plastic stage with obvious residual displacement and the reduction of stiffness. Due to the nonsymmetrical distribution of cracks appearing on the push and pull sides of the walls, the hysteretic curves were different regarding the negative displacement than regarding the positive displacement part of the loop. This became more visible on the curves after the ultimate load, for instance, for R-2 and M-2, due to the peeling of horizontal mortar joint or the broken of block. With the accumulation of plastic displacement, the walls progressed into the damage stage with reduced bearing capacity. With the developing of cracks, the envelope area of the hysteretic loop enlarged to absorb energy. At the last stage of the displacement control cycle, the hysteretic loop of specimens R-1 and R-2 moved from an arch shape to an S-shape, or even a Z-shape.

**Figure 6:**
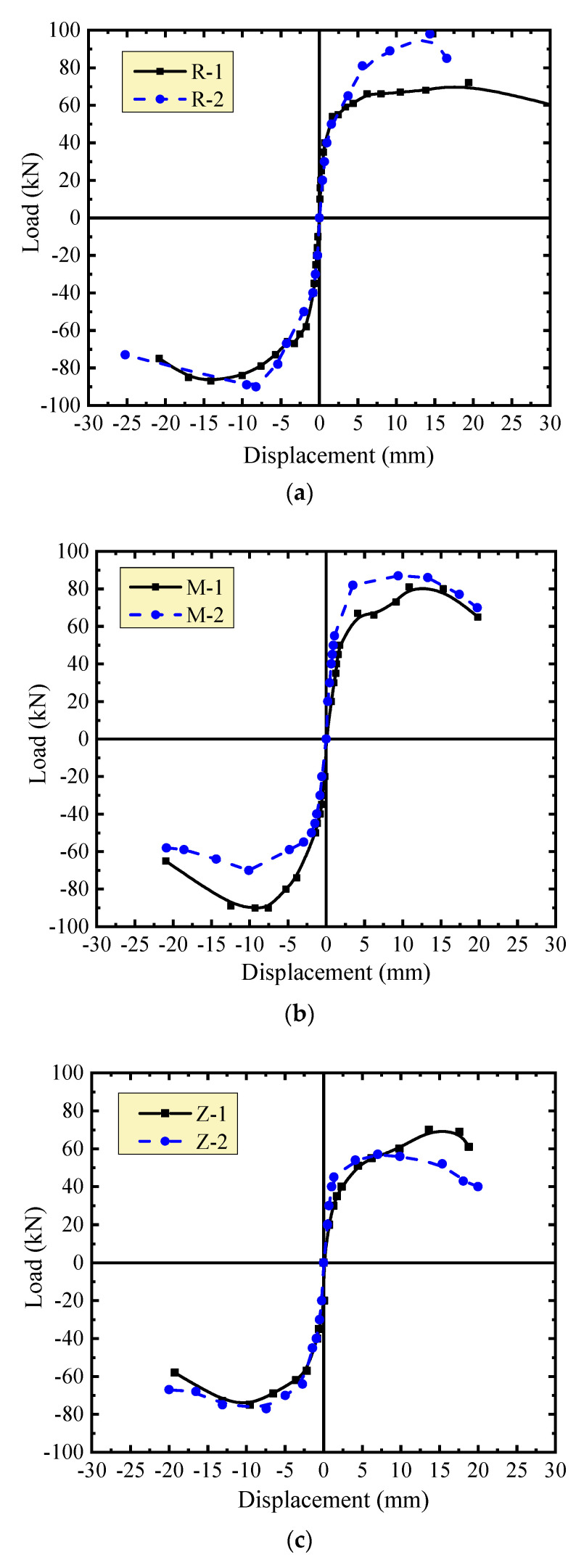
Comparison of envelop curves of the same wall under different vertical loads: (**a**) walls with insulation mortar joints; (**b**) walls with thin-layer mortar joints; (**c**) walls with vertical mineral wool joints.

The envelopes of hysteretic curves of the walls of each group are presented in Figure 6. The slope of the curves expresses the stiffness of the walls. Typically, the degradation of stiffness was clearly expressed with the increase of displacement. With smaller displacement under the same seismic load, greater stiffness of the walls was provided under the higher vertical compressive stress. This fits the normal regularity of the wall subjected to seismic loads as stated in specifications and previous studies [7,8,18,21]. After the peak-load, a larger displacement existed under the continued seismic load. This indicates a good deformability of the walls at damage states.

**Figure 7:**
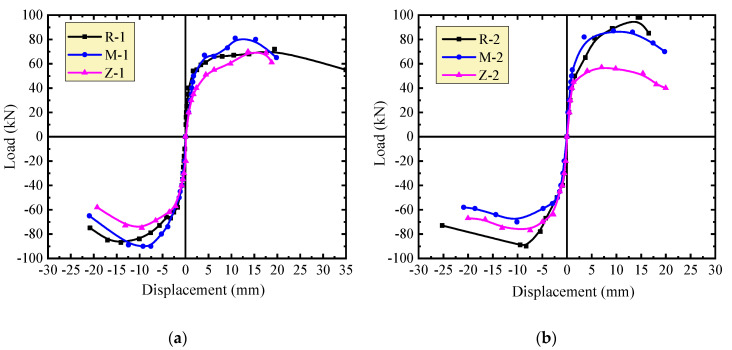
Comparison of envelope curves of different walls under the same vertical load: (a) *σ*_0_ = 0.3 MPa; (b) *σ*_0_ = 0.5 MPa.

To determine the effect of masonry joints on the stiffness of the walls, the envelopes of hysteretic curves of the walls under the same vertical compressive stress are presented in Figure 7. Before cracking, the walls had similar stiffness. After cracking, the stiffness changes of the walls M-1 and M-2 with thin-layer mortar joints were close to those of the walls R-1 and R-2 with self-insulation mortar joints. Under the vertical compressive stress *σ*_0_ = 0.3 MPa, the walls with thin-layer mortar joints had higher stiffness and better loading capacity than the walls with self-insulation mortar joints. However, under the vertical compressive stress *σ*_0_ = 0.5 MPa, this relationship changed to some extent. Clear changes of stiffness and loading capacity occurred on the walls Z-1 and Z-2 with vertical joints filled by mineral wool plates. Smaller stiffness and lower loading capacity of the walls were presented due to the unbound vertical joints.

The tested load and displacement at feature points of the hysteretic curves are listed in Table 2. The cracking load and displacement are those corresponding to the initial turning of the envelope curves. The ultimate load and displacement are the peak-load at push/pull envelope curve and the corresponding displacements. The damage loads are the 85% peak-loads at push/pull directions, and the corresponding displacements are the damage displacements. Generally, the walls with thin-layer mortar joints had higher cracking resistance about 13.1%, but ultimate capacity of about 4.5% lower than the walls with self-insulation mortar joints. The largest displacement at cracking appeared on the walls with thin-layer mortar joints, which was about 54.2% higher than that of the walls with self-insulation mortar joints. However, the best displacement ability was found on the walls with self-insulation mortar joints after the crack appeared. The cracking resistance and ultimate capacity of the walls with vertical joints filled by mineral wool plates were lowest, which were about 80.9% and 85.1% those of the walls with thin-layer mortar joints. At the same time, the displacement ability was also worst compared with the other walls.

With the increase of vertical compressive stress on top surface of the walls, the bearing capacity of the wall tended to be increased, while the displacement trended to be decreased. This is due to the friction on cracked sections increasing with higher compression, as seen in previous experimental results on AAC block walls [7,8].

### 3.3. Stiffness Degeneration

The secant stiffness *K*_n_ of the wall can be computed as follows [20],

(1) $$K_{n}=\frac{\left|+P_{n}\right|+\left|-P_{n}\right|}{\left|+\Delta_{n}\right|+\left|-\Delta_{n}\right|}$$

where *P*_n_ and *Δ*_n_ are the peak-load and the corresponding displacement at *n* cycle.

**Figure 8:**
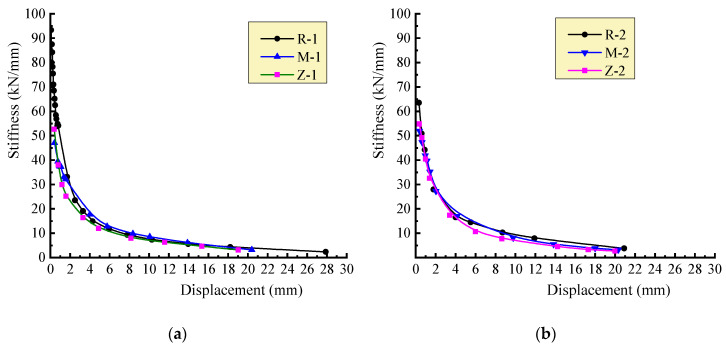
Stiffness degradation curve of each wall: (**a**) *σ*_0_ = 0.3 MPa; (**b**) *σ*_0_ = 0.5 MPa.

Hence, the stiffness degeneration curves are given out as presented in Figure 8. The walls with self-insulation mortar joints had the largest initial stiffness due to the high modulus of elasticity of the mortar compared to the AAC block [15]. A similar trend from fast to slow appeared on the curves. The fast degradation related to the appearance, extending and intersected developing of cracks. With the steady development of cracks until the ultimate load state, the stiffness degradation became slower. After that, the degradation reached a gentle stage. Relatively, due to the integrality of the walls weakened by the vertical joints filled by mineral wool plates, the walls had the largest stiffness degradation.

### 3.4. Energy Dissipation

**Figure 9:**
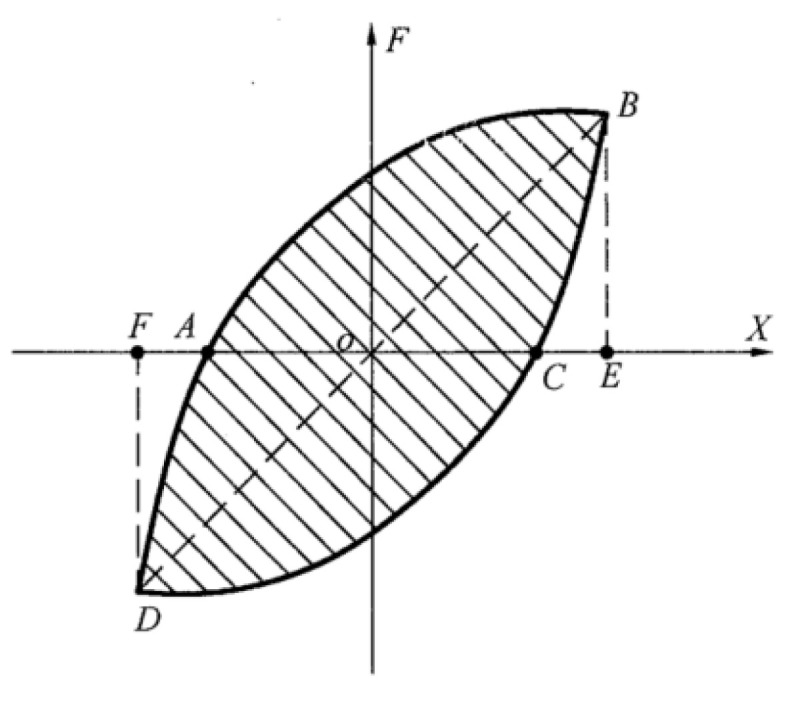
Energy dissipation computation.

The energy dissipation of the walls comprehensively reflects the bearing capacity with a rational displacement. This is commonly expressed as the envelope area of the hysteretic curves. As presented in Figure 9, the energy dissipating factor *φ* can be computed by Equation (2) [20]. The larger the factor *φ* is, the more energy is absorbed by the walls during the cyclic loading process. This means better energy dissipation and seismic resistance.

(2) $$\varphi =\frac{S_{\left(\mathrm{ABC}+\mathrm{CDA}\right)}}{S_{\left(\mathrm{OBE}+\mathrm{ODF}\right)}}$$

The equivalent viscous damping coefficient *ξ*_eq_ is also used to character the energy dissipation ability, as expressed by Equation (3). With a larger *ξ*_eq_, the wall has better energy dissipation.

(3) $$\xi_{\mathrm{eq}}=\varphi /2\pi$$

The computing results of the energy dissipation factor *φ* and the equivalent viscous damping coefficient *ξ*_eq_ are presented in Table 3. Comparatively, the walls with thin-layer mortar joints had best energy dissipation at cracking and ultimate states with larger values of *φ* and *ξ*_eq_. At cracking state and ultimate state, the average *φ* values of the walls with thin-layer mortar joints are 15.3% and 9.4% higher than those of the walls with insulation mortar joints, while the average *ξ*_eq_ values of the former are 16.7% and 10.5% higher than the later. This indicates that there was less confinement of the thin-layer mortar joints on the displacement of the walls. As a result, greater displacement occurred due to the smaller modulus of elasticity of the AAC blocks.

The values of *φ* and *ξ*_eq_ of the walls with vertical joints filled by mineral wool plates are similar to those of the walls with insulation mortar joints, while they are about 10–12% lower than those of the walls with thin-layer mortar joints. Meanwhile, higher vertical compressive stress led to a reduction of energy dissipation of the walls with vertical joints filled by mineral wool plates.

## 4. Prediction of Shear Resistance

The shear resistance of AAC insulation block walls are compared to the predictive values calculated by the formulas specified in current design codes for masonry walls. For convenience of explanation, the terms and symbols are unified in this paper.

The formula specified in China code JGJ/T17 for AAC walls is expressed as [21],

(4) $$V=0.75(f_{v}+0.2\sigma_{0})tl_{w}$$

where *V* is the shear resistance of the wall, *f*_v_ is the shear strength of the masonry along horizontal joint, *σ*_0_ is the vertical compressive stress on the wall, *t* and *l_w_* are the thickness and length of the wall.

The formula specified in China code GB50011 for concrete block masonry is expressed as [22],

(5) $$V=\left(f_{v}+0.66\mu \sigma_{0}\right)tl_{w}$$

(6) $$\mu =0.23-0.065\sigma_{0}/f_{c}$$

where *μ* is the factor considering the shear-compression effect on shear resistance, *f*_c_ is the compressive strength of the masonry.

Based on the specification of Eurocode 6 [23], the shear strength of the AAC wall can be calculated by Equation (6),

(7) $$V=f_{v}tl_{w}$$

Based on the mechanism of the crushing of the diagonal compressive strut, the shear resistance of AAC masonry wall specified in TMS 602-11 is calculated as follows [24],

(8) $$V=0.17f_{c}t\frac{hl_{w}{}^{2}}{h^{2}+{\left(\frac{3}{4}l_{w}\right)}^{2}}$$

where *h* is the effective height of the wall.

The comparison of test results in this study with the above equations are presented in Table 4. Higher predictive shear resistance of the walls is given by Equation (5). This is due to the different failure mechanism between the higher-strength concrete block wall and the lower-strength AAC block wall. For the Equations (4) and (7) using the shear strength of AAC block masonry along mortar joint, higher predictive shear resistances of the walls with self-insulation mortar joints, while lower predictive shear resistance of the walls with thin-layer mortar joints, are produced. This shows the necessity of strength matching between AAC block and mortar. However, Equations (4) and (7) give higher predictive shear resistance of the walls with vertical joints filled by mineral wool plates. This indicates the adverse effect of vertical joints without bonding together with mortar. The lowest predictive values by Equation (8) come from the lower compressive strength of the AAC self-insulation block, which has a limit of 3.45 MPa, specified in the code TMS 602-11 [24]. The slant compression strut was not able to be damaged in the walls of this study.

Generally, the shear resistance of the walls with thin-layer mortar joints can be conservatively predicted by Equations (4) and (7).

## 5. Conclusions

Based on the pseudo static test results of the six AAC self-insulation block walls, the conclusions can be drafted as follows:

(1) A typical damage pattern with intersected slant cracks was seen on the AAC self-insulation block walls under seismic loads. The type of mortar joints had some influence on the slant crack distribution. More almost-vertical cracks appeared on the walls with vertical joints filled by mineral wool plates. The blocks on push/pull sides of the walls tended to be easily broken under higher vertical compressive stress at failure state.
(2) The walls with thin-layer mortar joints had an entirely better seismic performance. The cracking resistance was about 13.1% higher with a displacement that was about 54.2% greater, in spite of lower ultimate capacity (about 4.5%) than the walls with self-insulation mortar joints. The cracking resistance and ultimate capacity of the walls with vertical joints filled by mineral wool plates were lowest with the worst displacement ability, which were about 80.9% and 85.1% of those of the walls with thin-layer mortar joints.
(3) The integrality of the walls was weakened with the vertical joints filled by mineral wool plates. This led to the reduction of seismic performance of the walls in stiffness and energy dissipation. Compared to the walls with thin-layer mortar joints, the walls with vertical joints filled by mineral wool plates underwent a reduction of about 10–12% in terms of the energy dissipation factor and the equivalent viscous damping coefficient.
(4) The vertical compressive stress had a certain impact on the seismic performance of the AAC self-insulation block walls. Under higher vertical compressive stress, the stiffness of the walls increased, and the energy dissipation decreased.
(5) With rational shear strength of the block masonry along the mortar joint, the shear resistance of the AAC self-insulation block masonry walls can be predicted by the formulas specified in China code JGJ/T17 and Eurocode 6.

## Figures and Tables

**Table 1:** Details of the specimens.

Wall Number	Dimension (mm)			Joint Thickness (mm)	Mineral Wool Vertical Joints	Vertical Compressive Stress (MPa)	Heat Transfer Coefficient [W/(m^2^·K)]
	Length	Height	Thickness				
R-1	1220	1245	250	10	no	0.3	0.541
R-2	1230	1240	250	10	no	0.5	0.522
M-1	1215	1220	250	5	no	0.3	0.508
M-2	1220	1230	250	5	no	0.5	0.514
Z-1	1230	1220	250	5	yes	0.3	0.524
Z-2	1230	1225	250	5	yes	0.5	0.541

**Table 2:** Tested load and displacement at feature points of hysteretic curves (push and pull).

Wall Number	Cracking		Ultimate		Damage	
	Load (kN)	Displacement (mm)	Load (kN)	Displacement (mm)	Load (kN)	Displacement (mm)
R-1	43	0.82	72	19.41	61	38.91
	43	0.77	87	14.09	74	20.51
R-2	50	1.58	98	14.36	83	16.23
	50	2.00	90	8.25	77	26.45
M-1	50	1.75	81	10.85	69	21.02
	50	1.36	90	9.27	77	24.67
M-2	55	1.10	87	9.42	74	20.89
	55	2.94	70	10.12	60	21.42
Z-1	40	2.35	70	13.62	60	18.33
	40	0.83	75	9.52	64	21.16
Z-2	45	1.33	57	7.02	48	24.18
	45	1.44	77	7.42	65	19.54

**Table 3:** Energy dissipation factor and equivalent viscous damping coefficient of the walls.

Wall Number	Cracking		Ultimate	
	*φ*	*ξ*_eq_	*φ*	*ξ*_eq_
R-1	1.83	0.29	1.73	0.27
R-2	1.96	0.31	1.87	0.30
M-1	2.24	0.36	1.80	0.29
M-2	2.13	0.34	2.14	0.34
Z-1	2.02	0.32	1.87	0.30
Z-2	1.86	0.30	1.69	0.27

**Table 4:** Comparison between tested and calculated results of shear resistance of the walls.

Wall Number	Tested (MPa)	Calculated by Equations (MPa)			
		(4)	(5)	(7)	(8)
R-1	79.5	98.4	157.0	112.8	61.9
R-2	94.0	108.4	156.9	113.8	61.9
M-1	85.5	70.6	120.1	75.9	66.0
M-2	78.5	80.1	119.3	76.3	66.4
Z-1	72.5	71.5	121.6	76.9	66.2
Z-2	67.0	80.7	157.1	76.9	66.4
